# Supplementary figures and images for: Molecular signatures of sexual communication in the phlebotomine sand flies
Source: PLoS Negl Trop Dis. 2020 Dec 28;14(12):e0008967. doi: 10.1371/journal.pntd.0008967 (PMC7793272; doi:10.1371/journal.pntd.0008967)

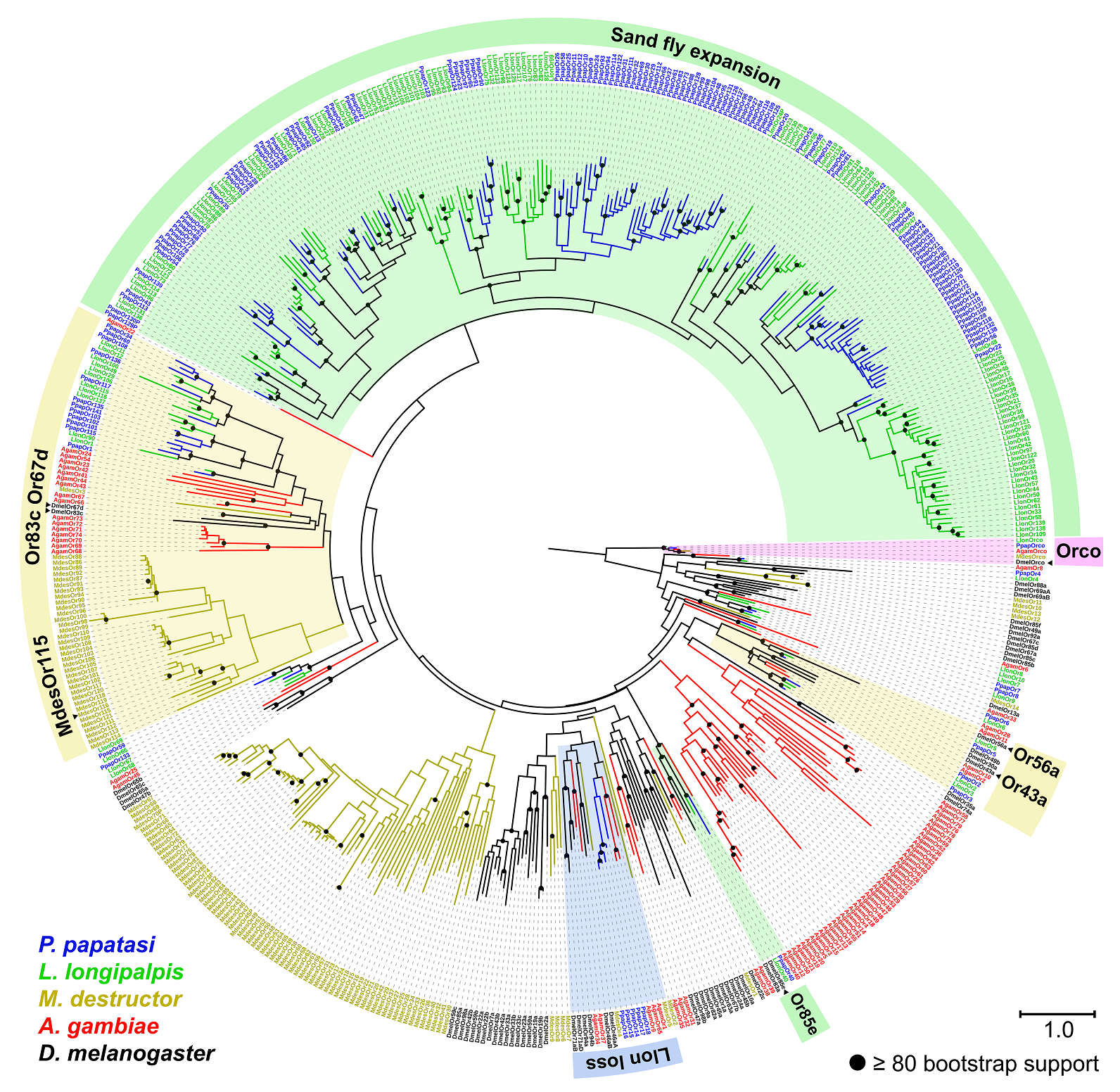

Supplement: S1 Fig — Expanded, conserved and lost OR lineages are shaded. The phylogeny was estimated using the JTT model of protein substitution and Maximum Likelihood method in RAxML v.8.2.4 [40]. The tree is rooted at the branch leading to Orco. Branch support based on 500 bootstrap replications. (TIF) [file pntd.0008967.s006.tif]

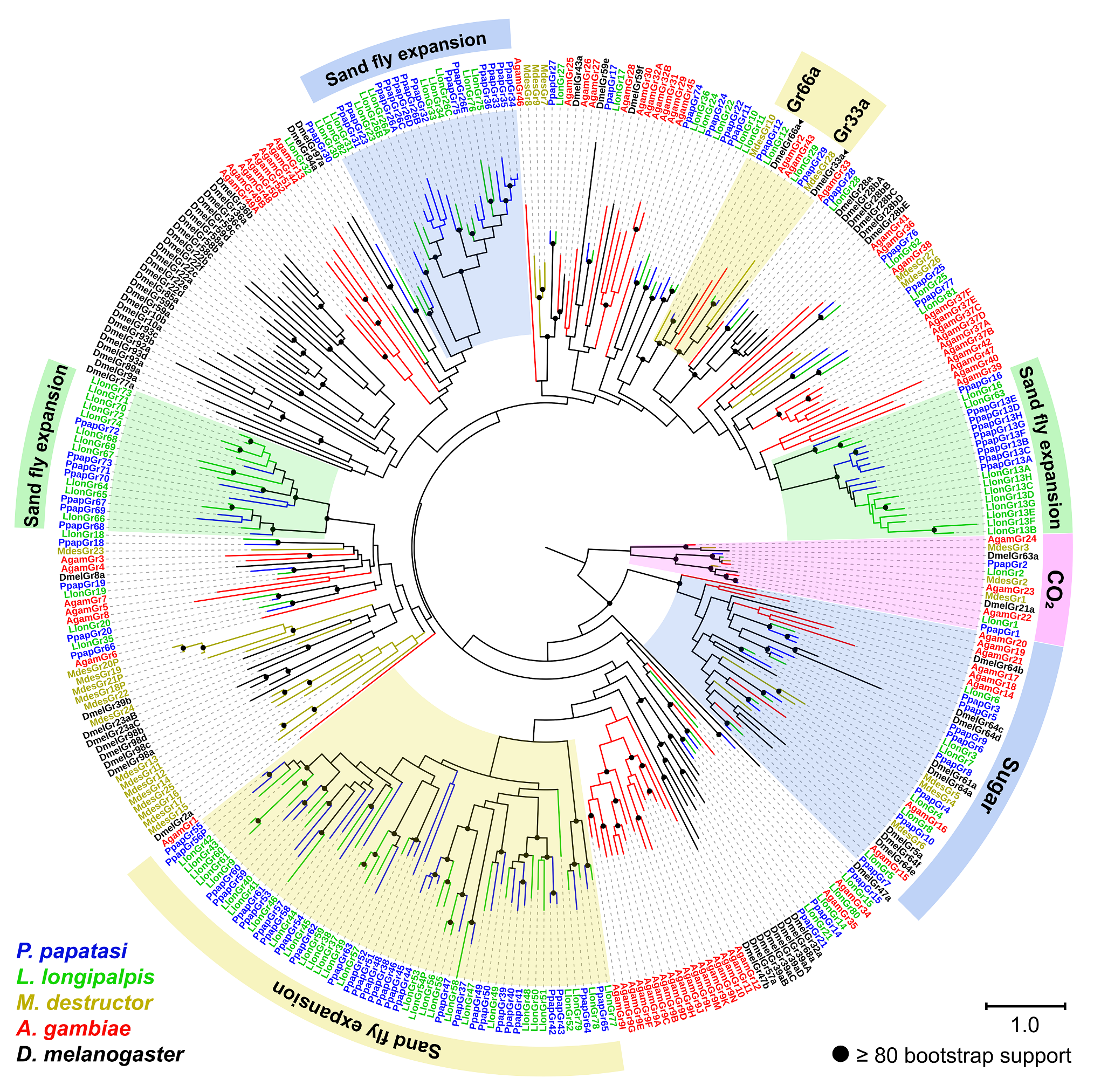

Supplement: S2 Fig — Expanded and conserved GR lineages are shaded. The phylogeny was estimated using the JTT model of protein substitution and Maximum Likelihood method in RAxML v.8.2.4 [40]. The tree is rooted at the branch leading to the CO2 receptors. Branch support based on 500 bootstrap replications. (TIF) [file pntd.0008967.s007.tif]

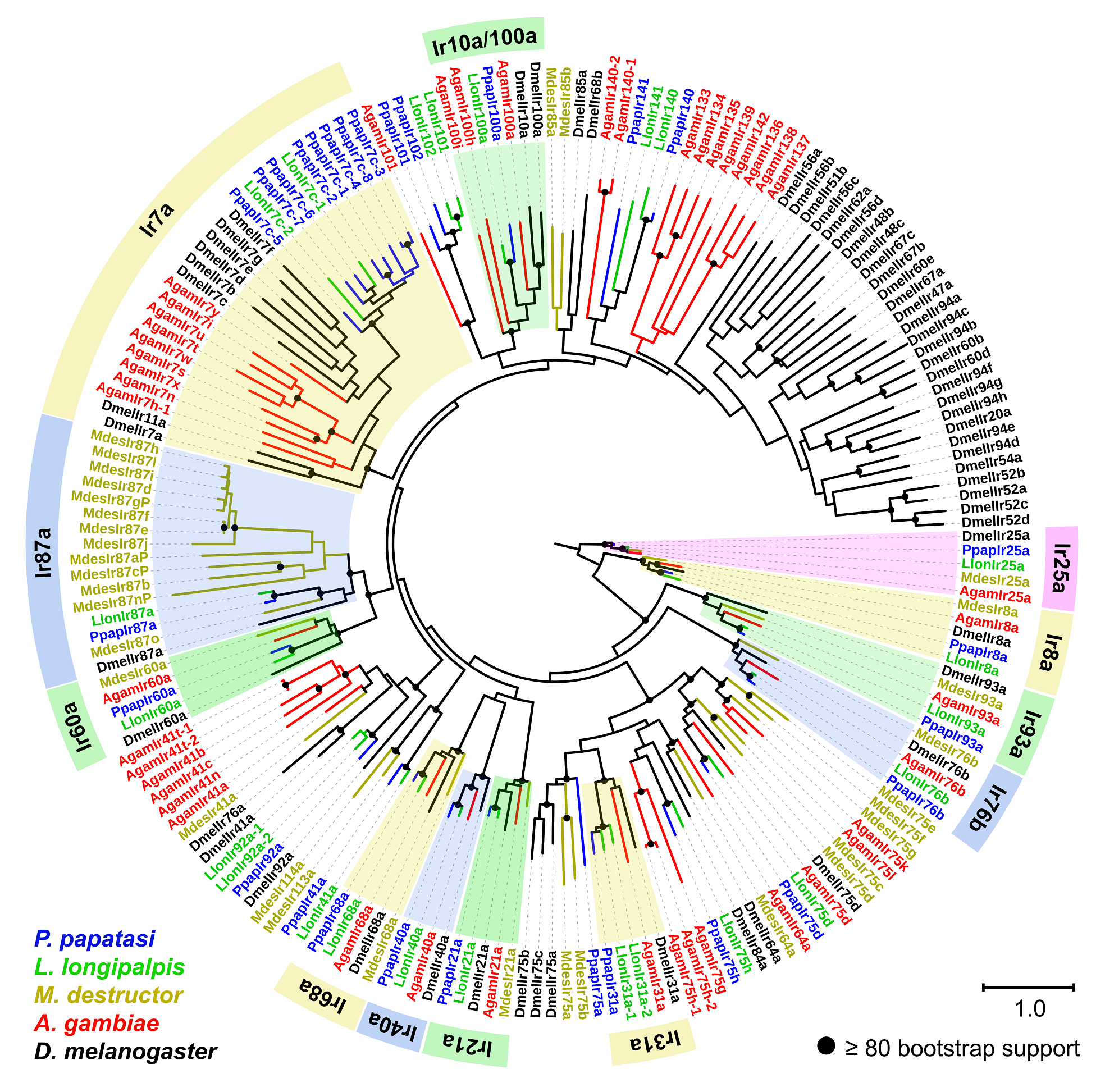

Supplement: S3 Fig — Conserved IR lineages are shaded. The phylogeny was estimated using the JTT model of protein substitution and Maximum Likelihood method in RAxML v.8.2.4 [40]. The tree is rooted at the branch leading to Ir25a and Ir8a. Branch support based on 500 bootstrap replications. (TIF) [file pntd.0008967.s008.tif]

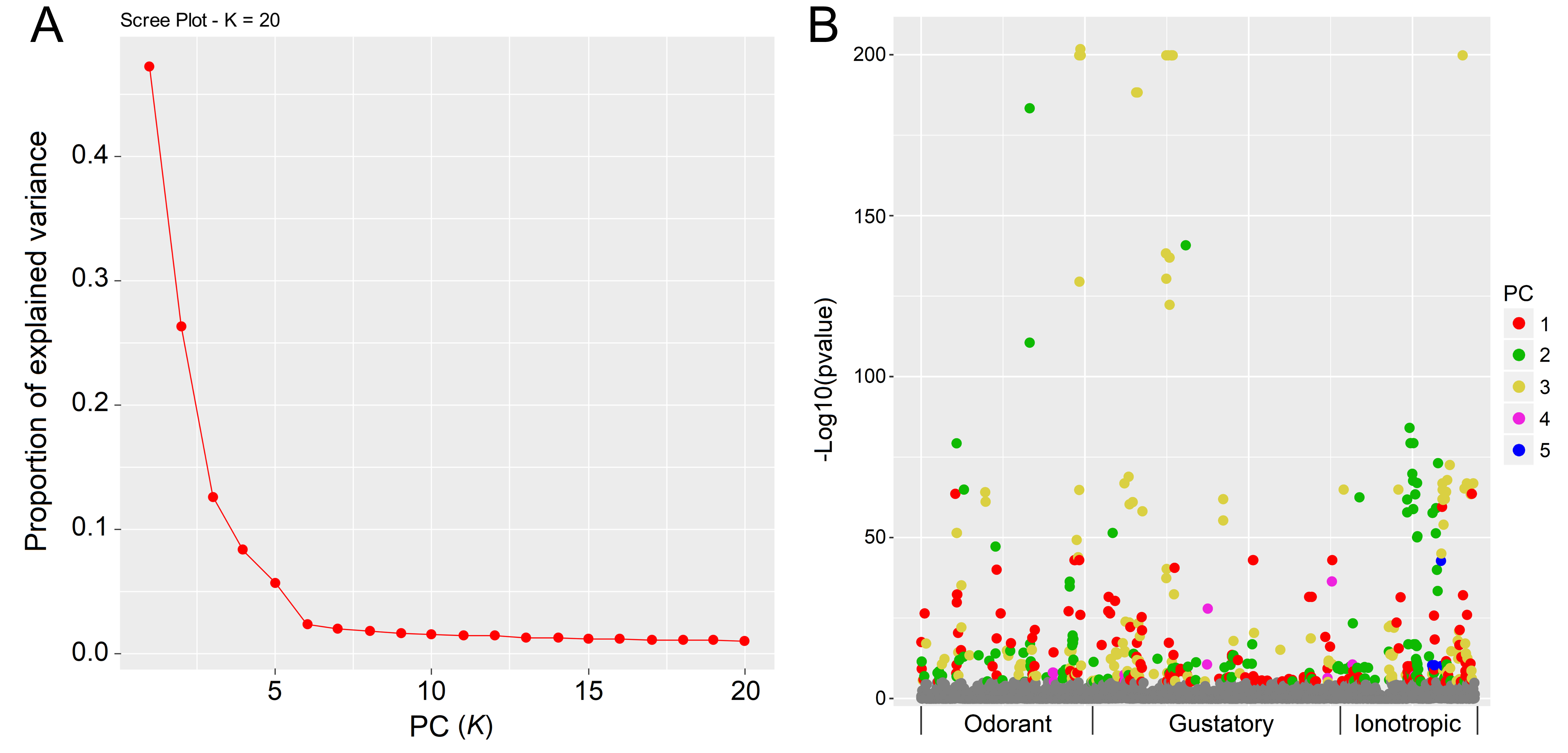

Supplement: S4 Fig — (A) An optimal number of PCs, K = 5, was selected following preliminary analysis with K = 20 and subsequent visualization of the scree plot. (B) Scatterplot showing the outliers after Bonferroni correction (0.05) and the PCs they are associated with based on component-wise analysis in PCadapt [46]. (TIF) [file pntd.0008967.s009.tif]

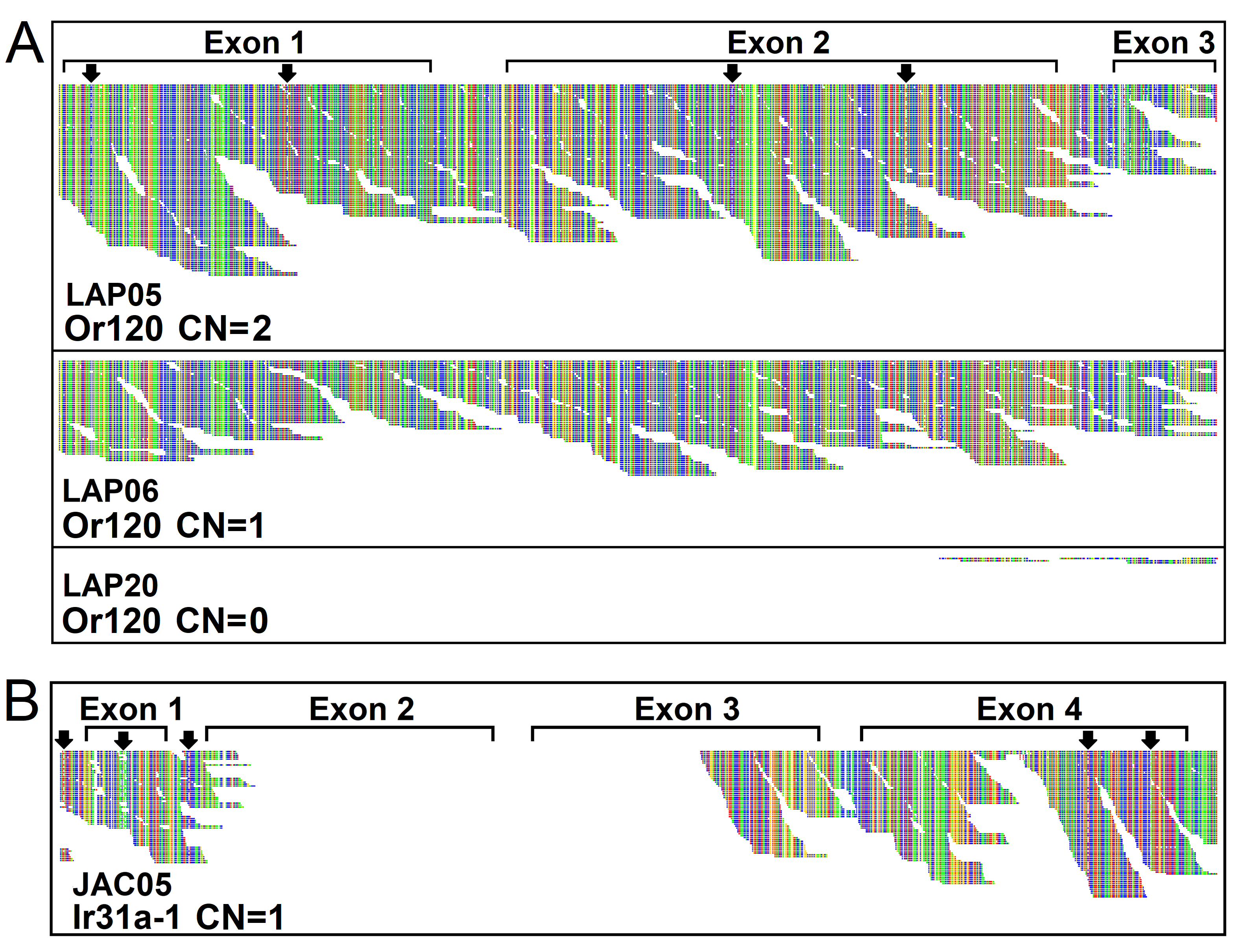

Supplement: S5 Fig — (A) Or120 has CN = 2 in LAP05, CN = 1 in LAP06 and CN = 0 in LAP20, suggesting the presence of intrapopulation variation in the form of two intact alleles (A+/+), an intact and a degraded allele (A+/-), and two degraded alleles (A-/-), respectively. Arrows indicate sites of heterozygosity in LAP05, while all sites are homozygous in LAP06. (B) Ir31a-1 in JAC05 illustrates the second situation where CN = 1 represents a moderately degraded pseudogene. Arrows indicate sites of heterozygosity indicating the parents likely had at least one allele with a similar degree of degradation. The Tablet software program [50] was used for visualization of read alignments. (TIF) [file pntd.0008967.s010.tif]
